# Bis{1-[(*E*)-(2-chloro­phen­yl)diazen­yl]naphthalen-2-olato}copper(II)

**DOI:** 10.1107/S1600536813016681

**Published:** 2013-06-22

**Authors:** Mohamed Amine Benaouida, Ali Benosmane, Hassiba Bouguerria, Salah Eddine Bouaoud, Hocine Merazig

**Affiliations:** a Unité de Recherche de Chimie de l’Environnement et Moléculaire Structurale, (CHEMS), Faculté des Sciences Exactes, Département de Chimie, Université Constantine 1, Algeria

## Abstract

The Cu^II^ atom in the title compound, [Cu(C_16_H_10_ClN_2_O)_2_], is located on an inversion center and is tetra­coordinated by two N and two O atoms from two bidentate 1-[(*E*)-(2-chloro­phen­yl)diazen­yl]naphthalen-2-olate ligands, forming a square-planar complex. In the crystal, mol­ecules are linked *via* weak C—H⋯O and C—H⋯Cl hydrogen bonds, forming chains propagating along [010]. There are also π–π inter­actions present involving adjacent naphthalene rings [centroid–centroid distance = 3.661 (13) Å].

## Related literature

For general background to azo compounds and their use in dyes, pigments and advanced materials, see: Lee *et al.* (2004 ▶); Oueslati *et al.* (2004 ▶). For related structures, see: Tai *et al.* (2010 ▶); Lin *et al.* (2010 ▶).
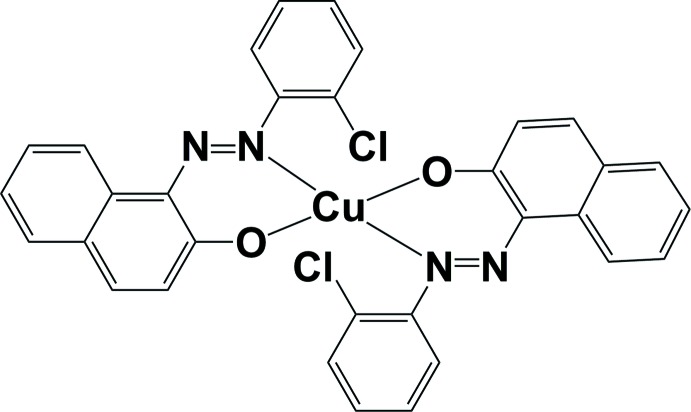


## Experimental

### 

#### Crystal data

[Cu(C_16_H_10_ClN_2_O)_2_]
*M* *_r_* = 626.99
Monoclinic, 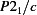

*a* = 10.2218 (4) Å
*b* = 7.8348 (3) Å
*c* = 17.5678 (6) Å
β = 111.941 (2)°
*V* = 1305.03 (9) Å^3^
*Z* = 2
Mo *K*α radiation
μ = 1.08 mm^−1^
*T* = 273 K
0.01 × 0.01 × 0.01 mm

#### Data collection

Bruker APEXII CCD diffractometer
7327 measured reflections
2299 independent reflections
1979 reflections with *I* > 2σ(*I*)
*R*_int_ = 0.030

#### Refinement

*R*[*F* ^2^ > 2σ(*F* ^2^)] = 0.034
*wR*(*F* ^2^) = 0.082
*S* = 1.04
2299 reflections
187 parameters
H-atom parameters constrained
Δρ_max_ = 0.48 e Å^−3^
Δρ_min_ = −0.28 e Å^−3^

### 

Data collection: *APEX2* (Bruker, 2006 ▶); cell refinement: *SAINT* (Bruker, 2006 ▶); data reduction: *SAINT*; program(s) used to solve structure: *SHELXS97* (Sheldrick, 2008 ▶); program(s) used to refine structure: *SHELXL97* (Sheldrick, 2008 ▶); molecular graphics: *ORTEP-3 for Windows* (Farrugia, 2012 ▶) and *Mercury* (Macrae *et al.*, 2008 ▶); software used to prepare material for publication: *WinGX* (Farrugia, 2012 ▶).

## Supplementary Material

Crystal structure: contains datablock(s) global, I. DOI: 10.1107/S1600536813016681/su2613sup1.cif

Structure factors: contains datablock(s) I. DOI: 10.1107/S1600536813016681/su2613Isup2.hkl

Additional supplementary materials:  crystallographic information; 3D view; checkCIF report

## Figures and Tables

**Table 1:** Hydrogen-bond geometry (Å, °)

*D*—H⋯*A*	*D*—H	H⋯*A*	*D*⋯*A*	*D*—H⋯*A*
C5—H5⋯O1^i^	0.93	2.62	3.300 (3)	130
C5—H5⋯Cl1^i^	0.93	2.94	3.682 (3)	138

Symmetry code: (i) 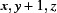.

**Fig. 1.**
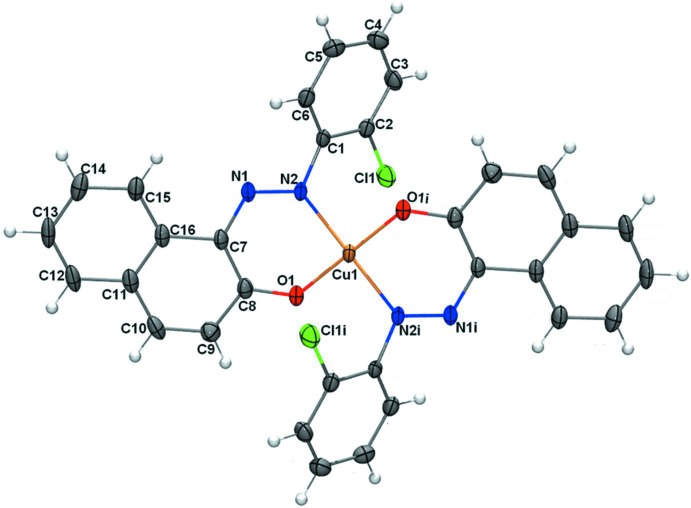
View of the molecular structure of the title compound, with atom labelling. Displacement ellipsoids are drawn at the 50% probability level [symmetry code: (i) -x+2, -y, -z+1]

**Fig. 2.**
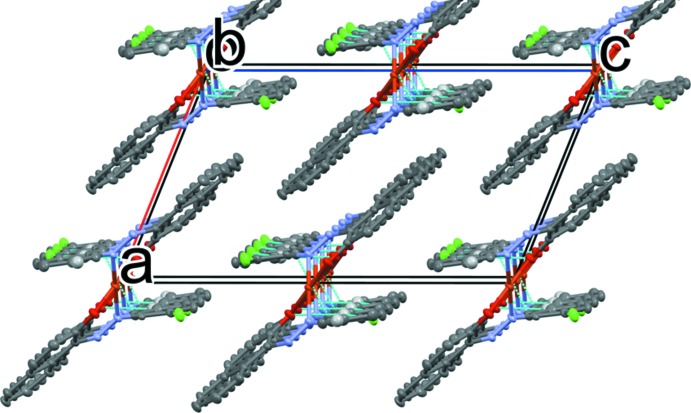
Partial view along the b axis of the crystal packing of the title compound, showing the hydrogen bonds as dashed lines (see Table 1 for details).

## supplementary crystallographic information

### Comment

Metal-complex dno's are coordination compounds in which a metal ion is linked to one or more ligands containing one or more electron-pair donors. Ligands with one and more donor groups are called mono-, di-, trifunctional ligands, *etc*. Coordination of two or more of the donor groups of such ligands to the same metal atom leads to di-, tri-, or tetradentate chelation, *etc*.; other names for these ligands are thus chelating agents or chelators. The metal complexes of these ligands are called chelates. The metals in metal-complex dno's are predominantly chromium and copper, and to a lesser extent cobalt, iron, and nickel. The ligand (*E*)-1-(*o*-tolyldiazenyl)naphthalen-2-ol, has been used previously to form complexes with Cu(OAc)_2_.H_2_O (Tai *et al.*, 2010) and Pd(OAc)_2_ (Lin *et al.*, 2010). Herein, we report of the crystal structure of a new copper complex of a similar ligand.

The title Cu^II^ complex (Fig. 1) contains two six-membered rings coordinated from two N,O-bidentate phenylazo-naphtholate ligands. It was found that the asymmetric unit contains one half molecule, the Cu atom lying on a centre of inversion. The Cu atom is tetra-coordinated with a normal square planar environment in which two N atoms and two O atoms are coplanar. The two N atoms and two O atoms around Cu atom are *trans* to each other with an O1—Cu1—N2 bond angle of 87.48 (8)° and O1—Cu1—N2^i^ angle of 92.52 (8)°; symmetry code: (i) (i) -*x*+2, -*y*, -*z*+1. The Cu1-O1 and Cu1-N2 bond distances are 1.8975 (17) Å and 1.961 (2) Å, respectively. The Cu1···Cl1 distances are 3.1525 (7) Å.

In the crystal, molecules are linked via weak C—H···O and C—H···Cl hydrogen bonds (Table 1) which form a one-dimensional chain running parallel to [010], as shown in Fig. 2. There are also π-π interactions present involving adjacent naphthalene rings with *Cg*1···*Cg*1^i^ = 3.661 (13) Å [*Cg*1 is the centroid of ring C7—C16; symmetry code: (i) *x*, *y* + 1, *z*].

### Experimental

A mixture of (*E*)-1-((2-chlorophenyl)diazenyl)naphthalen-2-ol (0.14 g, 0.5 mmol) and Cu(OAc)_2_.H_2_O (0.025 g, 0.25 mmol) was stirred at 293 K in methanol (10 ml) for 12 h. The mixture was filtered and set aside to crystallize at ambient temperature for three days, giving small block-like black crystals.

### Refinement

The C-bound H atoms were included in calculated positions and treated as riding atoms: C-H = 0.93 Å with U_iso_(H) = 1.2U_eq_(C). Despite a µ value = 1.08 mm^-1^ an absorption correction was not applied in view of the very small size of the crystal [0.01 × 0.01 × 0.01 mm].

### Crystal data

**Table:**

[Cu(C_16_H_10_ClN_2_O)_2_]	*Z* = 2
*M**_r_* = 626.99	*F*(000) = 638
Monoclinic, *P*2_1_/*c*	Least Squares Treatment of 25 SET4 setting angles.
Hall symbol: -P 2ybc	*D*_x_ = 1.595 Mg m^−^^3^
*a* = 10.2218 (4) Å	Mo *K*α radiation, λ = 0.71073 Å
*b* = 7.8348 (3) Å	µ = 1.08 mm^−^^1^
*c* = 17.5678 (6) Å	*T* = 273 K
β = 111.941 (2)°	Block, black
*V* = 1305.03 (9) Å^3^	0.01 × 0.01 × 0.01 mm

### Data collection

**Table:**

Bruker APEXII CCD diffractometer	1979 reflections with *I* > 2σ(*I*)
Radiation source: sealed tube	*R*_int_ = 0.030
Graphite monochromator	θ_max_ = 25.1°, θ_min_ = 2.6°
phi and ω scans	*h* = −11→12
7327 measured reflections	*k* = −9→9
2299 independent reflections	*l* = −20→20

### Refinement

**Table:**

Refinement on *F*^2^	Primary atom site location: structure-invariant direct methods
Least-squares matrix: full	Secondary atom site location: difference Fourier map
*R*[*F*^2^ > 2σ(*F*^2^)] = 0.034	Hydrogen site location: inferred from neighbouring sites
*wR*(*F*^2^) = 0.082	H-atom parameters constrained
*S* = 1.04	*w* = 1/[σ^2^(*F*_o_^2^) + (0.0406*P*)^2^ + 0.9147*P*] where *P* = (*F*_o_^2^ + 2*F*_c_^2^)/3
2299 reflections	(Δ/σ)_max_ < 0.001
187 parameters	Δρ_max_ = 0.48 e Å^−^^3^
0 restraints	Δρ_min_ = −0.28 e Å^−^^3^

### Special details

**Table:**

Geometry. Bond distances, angles *etc*. have been calculated using the rounded fractional coordinates. All su's are estimated from the variances of the (full) variance-covariance matrix. The cell e.s.d.'s are taken into account in the estimation of distances, angles and torsion angles
Refinement. Refinement on *F*^2^ for ALL reflections except those flagged by the user for potential systematic errors. Weighted *R*-factors *wR* and all goodnesses of fit *S* are based on *F*^2^, conventional *R*-factors *R* are based on *F*, with *F* set to zero for negative *F*^2^. The observed criterion of *F*^2^ > σ(*F*^2^) is used only for calculating -*R*-factor-obs *etc*. and is not relevant to the choice of reflections for refinement. *R*-factors based on *F*^2^ are statistically about twice as large as those based on *F*, and *R*-factors based on ALL data will be even larger.

### Fractional atomic coordinates and isotropic or equivalent isotropic displacement parameters (Å^2^)

**Table:**

	*x*	*y*	*z*	*U*_iso_*/*U*_eq_	
Cu1	1.00000	0.00000	0.50000	0.0201 (1)	
Cl1	0.86221 (8)	0.03646 (8)	0.30754 (4)	0.0321 (2)	
O1	0.87383 (17)	−0.1495 (2)	0.52340 (10)	0.0247 (6)	
N1	0.7529 (2)	0.1928 (3)	0.49516 (12)	0.0215 (6)	
N2	0.8458 (2)	0.1667 (3)	0.46293 (12)	0.0211 (6)	
C1	0.8501 (2)	0.2972 (3)	0.40751 (15)	0.0214 (7)	
C2	0.8675 (3)	0.2510 (3)	0.33477 (15)	0.0232 (8)	
C3	0.8873 (3)	0.3725 (4)	0.28366 (16)	0.0282 (8)	
C4	0.8913 (3)	0.5422 (4)	0.30481 (17)	0.0310 (9)	
C5	0.8705 (3)	0.5902 (3)	0.37502 (16)	0.0291 (8)	
C6	0.8498 (3)	0.4678 (3)	0.42576 (16)	0.0263 (8)	
C7	0.7306 (2)	0.0731 (3)	0.54482 (15)	0.0207 (7)	
C8	0.7818 (3)	−0.0968 (3)	0.55231 (15)	0.0219 (7)	
C9	0.7262 (3)	−0.2196 (3)	0.59274 (16)	0.0274 (8)	
C10	0.6318 (3)	−0.1733 (4)	0.62659 (16)	0.0304 (9)	
C11	0.5848 (3)	−0.0025 (4)	0.62456 (16)	0.0267 (8)	
C12	0.4917 (3)	0.0451 (4)	0.66382 (16)	0.0317 (9)	
C13	0.4493 (3)	0.2096 (4)	0.66213 (17)	0.0360 (10)	
C14	0.4949 (3)	0.3334 (4)	0.62085 (17)	0.0345 (9)	
C15	0.5854 (3)	0.2909 (4)	0.58222 (16)	0.0286 (8)	
C16	0.6323 (2)	0.1228 (3)	0.58341 (15)	0.0227 (8)	
H3	0.89790	0.34050	0.23530	0.0340*	
H4	0.90820	0.62490	0.27160	0.0370*	
H5	0.87050	0.70520	0.38810	0.0350*	
H6	0.83550	0.50100	0.47290	0.0310*	
H9	0.75520	−0.33280	0.59580	0.0330*	
H10	0.59690	−0.25620	0.65190	0.0360*	
H12	0.45960	−0.03700	0.69090	0.0380*	
H13	0.38930	0.23990	0.68870	0.0430*	
H14	0.46410	0.44550	0.61940	0.0410*	
H15	0.61560	0.37480	0.55510	0.0340*	

### Atomic displacement parameters (Å^2^)

**Table:**

	*U*^11^	*U*^22^	*U*^33^	*U*^12^	*U*^13^	*U*^23^
Cu1	0.0171 (2)	0.0261 (2)	0.0214 (2)	0.0055 (2)	0.0123 (2)	0.0032 (2)
Cl1	0.0429 (4)	0.0302 (4)	0.0314 (4)	0.0010 (3)	0.0235 (3)	−0.0026 (3)
O1	0.0221 (9)	0.0280 (10)	0.0301 (10)	0.0037 (8)	0.0169 (8)	0.0024 (8)
N1	0.0169 (10)	0.0304 (12)	0.0199 (10)	0.0027 (9)	0.0101 (9)	0.0008 (9)
N2	0.0183 (10)	0.0276 (11)	0.0220 (11)	0.0058 (9)	0.0129 (9)	0.0027 (9)
C1	0.0155 (12)	0.0287 (13)	0.0224 (13)	0.0061 (10)	0.0099 (11)	0.0058 (11)
C2	0.0205 (13)	0.0276 (14)	0.0233 (13)	0.0049 (11)	0.0104 (11)	0.0016 (11)
C3	0.0289 (15)	0.0391 (16)	0.0200 (13)	0.0039 (12)	0.0131 (12)	0.0059 (12)
C4	0.0313 (15)	0.0342 (16)	0.0279 (15)	0.0045 (12)	0.0116 (13)	0.0109 (12)
C5	0.0294 (15)	0.0260 (14)	0.0306 (15)	0.0067 (12)	0.0096 (13)	0.0055 (12)
C6	0.0242 (13)	0.0326 (15)	0.0246 (14)	0.0090 (11)	0.0121 (12)	0.0033 (11)
C7	0.0139 (12)	0.0304 (13)	0.0190 (12)	−0.0001 (11)	0.0074 (10)	−0.0008 (11)
C8	0.0153 (12)	0.0322 (14)	0.0181 (12)	0.0000 (11)	0.0062 (10)	−0.0008 (11)
C9	0.0265 (14)	0.0269 (14)	0.0297 (14)	−0.0013 (11)	0.0117 (12)	0.0000 (12)
C10	0.0288 (15)	0.0426 (16)	0.0252 (14)	−0.0069 (13)	0.0164 (12)	0.0021 (13)
C11	0.0185 (12)	0.0432 (16)	0.0201 (12)	−0.0012 (12)	0.0091 (11)	−0.0018 (13)
C12	0.0232 (14)	0.0547 (19)	0.0226 (14)	−0.0019 (13)	0.0147 (12)	−0.0012 (13)
C13	0.0254 (15)	0.060 (2)	0.0304 (15)	0.0014 (14)	0.0193 (13)	−0.0092 (15)
C14	0.0283 (15)	0.0435 (17)	0.0368 (16)	0.0055 (13)	0.0179 (13)	−0.0092 (14)
C15	0.0231 (14)	0.0393 (16)	0.0274 (14)	0.0040 (12)	0.0139 (12)	−0.0019 (13)
C16	0.0150 (12)	0.0364 (15)	0.0176 (12)	−0.0006 (11)	0.0070 (11)	−0.0040 (11)

### Geometric parameters (Å, º)

**Table:**

Cu1—Cl1	3.1525 (7)	C8—C9	1.434 (4)
Cu1—O1	1.8975 (17)	C9—C10	1.358 (4)
Cu1—N2	1.961 (2)	C10—C11	1.418 (4)
Cu1—Cl1^i^	3.1525 (7)	C11—C12	1.418 (4)
Cu1—O1^i^	1.8975 (17)	C11—C16	1.409 (4)
Cu1—N2^i^	1.961 (2)	C12—C13	1.357 (4)
Cl1—C2	1.743 (2)	C13—C14	1.392 (4)
O1—C8	1.292 (4)	C14—C15	1.377 (4)
N1—N2	1.291 (3)	C15—C16	1.399 (4)
N1—C7	1.357 (3)	C3—H3	0.9300
N2—C1	1.424 (3)	C4—H4	0.9300
C1—C2	1.402 (4)	C5—H5	0.9300
C1—C6	1.375 (3)	C6—H6	0.9300
C2—C3	1.375 (4)	C9—H9	0.9300
C3—C4	1.377 (4)	C10—H10	0.9300
C4—C5	1.380 (4)	C12—H12	0.9300
C5—C6	1.378 (4)	C13—H13	0.9300
C7—C8	1.418 (3)	C14—H14	0.9300
C7—C16	1.459 (3)	C15—H15	0.9300
			
Cl1—Cu1—O1	102.76 (5)	O1—C8—C9	117.5 (2)
Cl1—Cu1—N2	66.55 (6)	C7—C8—C9	118.4 (3)
Cl1—Cu1—Cl1^i^	180.00	C8—C9—C10	121.0 (2)
Cl1—Cu1—O1^i^	77.24 (5)	C9—C10—C11	122.0 (3)
Cl1—Cu1—N2^i^	113.45 (6)	C10—C11—C12	121.2 (3)
O1—Cu1—N2	87.48 (8)	C10—C11—C16	119.5 (3)
Cl1^i^—Cu1—O1	77.24 (5)	C12—C11—C16	119.3 (3)
O1—Cu1—O1^i^	180.00	C11—C12—C13	120.4 (3)
O1—Cu1—N2^i^	92.52 (8)	C12—C13—C14	120.5 (3)
Cl1^i^—Cu1—N2	113.45 (6)	C13—C14—C15	120.4 (3)
O1^i^—Cu1—N2	92.52 (8)	C14—C15—C16	120.7 (3)
N2—Cu1—N2^i^	180.00	C7—C16—C11	118.8 (2)
Cl1^i^—Cu1—O1^i^	102.76 (5)	C7—C16—C15	122.4 (2)
Cl1^i^—Cu1—N2^i^	66.55 (6)	C11—C16—C15	118.8 (2)
O1^i^—Cu1—N2^i^	87.48 (8)	C2—C3—H3	120.00
Cu1—Cl1—C2	80.64 (8)	C4—C3—H3	120.00
Cu1—O1—C8	122.78 (15)	C3—C4—H4	120.00
N2—N1—C7	120.0 (2)	C5—C4—H4	120.00
Cu1—N2—N1	126.26 (17)	C4—C5—H5	120.00
Cu1—N2—C1	118.61 (16)	C6—C5—H5	120.00
N1—N2—C1	113.7 (2)	C1—C6—H6	120.00
N2—C1—C2	119.0 (2)	C5—C6—H6	120.00
N2—C1—C6	122.4 (2)	C8—C9—H9	119.00
C2—C1—C6	118.4 (2)	C10—C9—H9	120.00
Cl1—C2—C1	119.85 (19)	C9—C10—H10	119.00
Cl1—C2—C3	119.1 (2)	C11—C10—H10	119.00
C1—C2—C3	121.1 (2)	C11—C12—H12	120.00
C2—C3—C4	119.3 (3)	C13—C12—H12	120.00
C3—C4—C5	120.4 (3)	C12—C13—H13	120.00
C4—C5—C6	120.0 (2)	C14—C13—H13	120.00
C1—C6—C5	120.8 (2)	C13—C14—H14	120.00
N1—C7—C8	124.3 (2)	C15—C14—H14	120.00
N1—C7—C16	115.1 (2)	C14—C15—H15	120.00
C8—C7—C16	120.1 (2)	C16—C15—H15	120.00
O1—C8—C7	124.1 (2)		
			
O1—Cu1—Cl1—C2	123.11 (12)	N2—C1—C6—C5	172.4 (3)
N2—Cu1—Cl1—C2	41.58 (13)	C2—C1—C6—C5	−2.0 (4)
O1^i^—Cu1—Cl1—C2	−56.89 (12)	Cl1—C2—C3—C4	179.9 (2)
N2^i^—Cu1—Cl1—C2	−138.42 (13)	C1—C2—C3—C4	0.6 (5)
Cl1—Cu1—O1—C8	−102.24 (18)	C2—C3—C4—C5	−2.4 (5)
N2—Cu1—O1—C8	−36.98 (19)	C3—C4—C5—C6	2.0 (5)
Cl1^i^—Cu1—O1—C8	77.76 (18)	C4—C5—C6—C1	0.3 (5)
N2^i^—Cu1—O1—C8	143.03 (19)	N1—C7—C8—O1	11.7 (4)
Cl1—Cu1—N2—N1	142.2 (2)	N1—C7—C8—C9	−166.8 (2)
Cl1—Cu1—N2—C1	−52.32 (16)	C16—C7—C8—O1	−176.7 (2)
O1—Cu1—N2—N1	37.1 (2)	C16—C7—C8—C9	4.8 (4)
O1—Cu1—N2—C1	−157.39 (18)	N1—C7—C16—C11	169.5 (2)
Cl1^i^—Cu1—N2—N1	−37.8 (2)	N1—C7—C16—C15	−11.7 (3)
Cl1^i^—Cu1—N2—C1	127.68 (16)	C8—C7—C16—C11	−2.9 (4)
O1^i^—Cu1—N2—N1	−142.9 (2)	C8—C7—C16—C15	176.0 (2)
O1^i^—Cu1—N2—C1	22.61 (18)	O1—C8—C9—C10	178.3 (2)
Cu1—Cl1—C2—C1	−33.3 (2)	C7—C8—C9—C10	−3.1 (4)
Cu1—Cl1—C2—C3	147.4 (3)	C8—C9—C10—C11	−0.7 (4)
Cu1—O1—C8—C7	21.9 (3)	C9—C10—C11—C12	−176.8 (3)
Cu1—O1—C8—C9	−159.62 (18)	C9—C10—C11—C16	2.7 (4)
C7—N1—N2—Cu1	−18.4 (3)	C10—C11—C12—C13	179.1 (3)
C7—N1—N2—C1	175.5 (2)	C16—C11—C12—C13	−0.3 (4)
N2—N1—C7—C8	−13.2 (4)	C10—C11—C16—C7	−0.8 (4)
N2—N1—C7—C16	174.8 (2)	C10—C11—C16—C15	−179.8 (3)
Cu1—N2—C1—C2	53.1 (3)	C12—C11—C16—C7	178.6 (2)
Cu1—N2—C1—C6	−121.3 (2)	C12—C11—C16—C15	−0.3 (4)
N1—N2—C1—C2	−139.7 (2)	C11—C12—C13—C14	1.0 (4)
N1—N2—C1—C6	46.0 (3)	C12—C13—C14—C15	−1.0 (4)
N2—C1—C2—Cl1	7.7 (3)	C13—C14—C15—C16	0.4 (4)
N2—C1—C2—C3	−173.0 (3)	C14—C15—C16—C7	−178.6 (3)
C6—C1—C2—Cl1	−177.7 (2)	C14—C15—C16—C11	0.3 (4)
C6—C1—C2—C3	1.6 (4)		

Symmetry code: (i) −*x*+2, −*y*, −*z*+1.

### Hydrogen-bond geometry (Å, º)

**Table:**

*D*—H···*A*	*D*—H	H···*A*	*D*···*A*	*D*—H···*A*
C5—H5···O1^ii^	0.93	2.62	3.300 (3)	130
C5—H5···Cl1^ii^	0.93	2.94	3.682 (3)	138

Symmetry code: (ii) *x*, *y*+1, *z*.
